# CREB: A Promising Therapeutic Target for Treating Psychiatric Disorders

**DOI:** 10.2174/1570159X22666240206111838

**Published:** 2024-07-03

**Authors:** Wei Guan, Mei-Xin Ni, Hai-Juan Gu, Yang Yang

**Affiliations:** 1 Department of Pharmacology, Pharmacy College, Nantong University, Nantong 226001, Jiangsu, China;; 2 Department of Pharmacy, Affiliated Tumor Hospital of Nantong University/Nantong Tumor Hospital, Nantong, Jiangsu 226361, China

**Keywords:** CREB, depression, human diseases, expression levels, target gene, psychiatric disorders

## Abstract

Psychiatric disorders are complex, multifactorial illnesses. It is challenging for us to understand the underlying mechanism of psychiatric disorders. In recent years, the morbidity of psychiatric disorders has increased yearly, causing huge economic losses to the society. Although some progress, such as psychotherapy drugs and electroconvulsive therapy, has been made in the treatment of psychiatric disorders, including depression, anxiety, bipolar disorder, obsessive-compulsive and autism spectrum disorders, antidepressants and psychotropic drugs have the characteristics of negative effects and high rate of relapse. Therefore, researchers continue to seek suitable interventions. cAMP response element binding protein (CREB) belongs to a protein family and is widely distributed in the majority of brain cells that function as a transcription factor. It has been demonstrated that CREB plays an important role in neurogenesis, synaptic plasticity, and neuronal growth. This review provides a 10-year update of the 2013 systematic review on the multidimensional roles of CREB-mediated transcriptional signaling in psychiatric disorders. We also summarize the classification of psychiatric disorders and elucidate the involvement of CREB and related downstream signalling pathways in psychiatric disorders. Importantly, we analyse the CREB-related signal pathways involving antidepressants and antipsychotics to relieve the pathological process of psychiatric disorders. This review emphasizes that CREB signalling may have a vast potential to treat psychiatric disorders like depression. Furthermore, it would be helpful for the development of potential medicine to make up for the imperfection of current antidepressants and antipsychotics.

## 1 INTRODUCTION

**Fig. (1):**
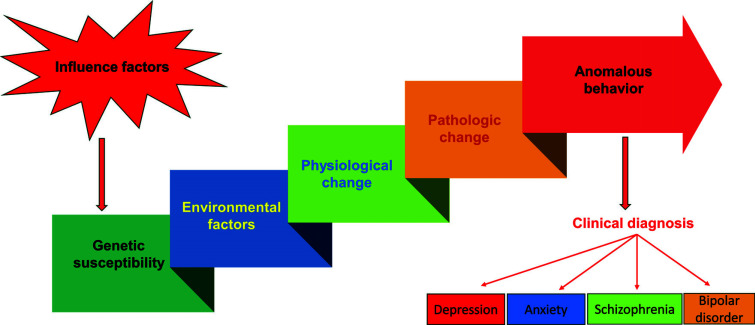
Psychiatric disorders are the comprehensive results of genetic susceptibility, environmental factors, and clinical manifestation.

Psychiatric disorders, such as anxiety, depression, and obsessive-compulsive disorder, are a large group of common brain disorders involving complex disturbances in social-cognitive functioning [1], which cause immense suffering and carry enormous consequences for societies and economies. According to the World Health Organization, psychiatric disorders affected 792 million people around the world in 2021, accounting for over 10 percent of the world's population [2]. Studies have shown that psychiatric disorders are caused by multiple factors, such as psychological, physiological, and social elements (Fig. **1**). Hereditary factors, external pressure stimulus, smoking and drinking, as well as chronic diseases, are the main causes of psychiatric disorders [3]. The COVID-19 pandemic could further exacerbate the threat of psychiatric disorders in terms of suicide risk [4]. Furthermore, compared to males, females have a high prevalence and greater pressure on living quality. Available data shows that the prevalence of depression and anxiety is several times higher in women than in men, while the prevalence of suicidal ideation and suicide death was found to be higher in males with bipolar disorder and schizophrenia [5]. There are indications that the treatment for psychiatric disorders is inevitable.

At the moment, the pathophysiology and molecular mechanisms causing psychiatric disorders are not very clear, and early diagnosis and pharmacological treatments still confront difficulties. In actual clinical practice, psychotherapy and only limited antidepressants and antipsychotics are available for psychiatric disorders [6, 7]. Many more problems have come to the surface, although we have made much progress. First, many patients with psychiatric disorders do not respond to pharmacological treatment. Second, most of the antipsychotics approved by the Food and Drug Administration (FDA) exhibit healing efficacy by targeting only a small minority of molecular targets, such as type 2 dopaminergic receptor (DRD2) [8, 9]. This is also the factor leading to drug discovery for psychiatric disorders gaining little progress for decades [10, 11]. Finally, antipsychotics can cause serious side effects, including weight gain, dysphoria and hypertension [12]. Therefore, it is essential to seek the possible therapeutic target.

**Fig. (2):**
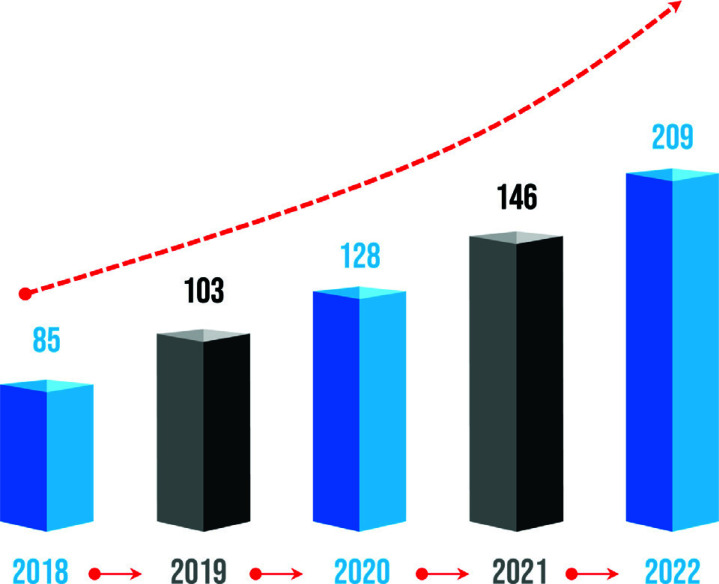
The study on CREB in psychiatric disorders has been increasing every year.

Several studies have shown that transcription factor CREB plays a critical role in brain development and neurogenesis [13, 14]. CREB is an important molecular target underlying neuronal differentiation, synaptic plasticity, learning and memory [15]. Moreover, the expression of CREB was significantly decreased in patients with depression than in the healthy controls [16]. Similarly, the low level of CREB protein was seen in the methamphetamine-induced anxiety and depression model of rats [14]. The main form of CREB with bioactivity is a phosphorylated state, and multiple protein kinases convert CREB to its activated form [17, 18]. CREB is the transcription factor of brain-derived neurotrophic factor (BDNF), thereby facilitating the recruitment of BDNF to its promoter [19]. A growing body of research demonstrated that the CREB/BDNF signalling pathway participates in the development of nerve growth and plays important roles in psychiatric disorders, such as depression, anxiety, and bipolar disorder (Fig. **2**) [20-22].

In this review, we first expound on the classifications of psychiatric disorders and further clarify the involved mechanism of CREB and its downstream paths in psychiatric disorders. Moreover, we also summarize the CREB involved in some antidepressants and antipsychotics with excellent treatment effects in psychiatric disorders. The findings of the present review make a better understanding of psychiatric disorders and the mechanisms involved in the development of promising therapeutic strategies in psychiatric disorders.

## 2 METHODS

This review was conducted first by defining a theme, then searching literature and assessing data eligibility, and finally with the presentation of the results. We manually reviewed articles from March, 2012 to December, 2022 employing PubMed, Medline, Embase, Google Scholar and the Cochrane Database with the following search terms: “psychiatric disorders”, AND “CREB”, AND “mechanisms”, OR “signal pathway”, AND “depression”, OR “anxiety”, OR “BDNF”, OR “human diseases”, OR “schizophrenia”, OR “bipolar disorder”. We also reviewed articles obtained in the initial search as references cited in manuscripts. The search strategy in our review was consistent with previously published reviews on depression or other neuropsychiatric disorders. Fig. (**3**) shows the screening process of articles. Finally, we included 180 articles as the theoretical basis of psychiatric disorders after the exclusion of the substandard articles.

The basic condition of the literature search is that the language of all included studies should be written in English, and the eligibility criteria for inclusion in the review were determined by browsing the titles and abstracts of the articles. Given the breadth and specificity of the topic, studies on Alzheimer's disease (AD), autism and Asperger's syndrome were not included in the search terms and were not discussed in this review.

The inclusion criteria should include the following sections: 1) the theme of the articles was the roles of CREB in psychiatric disorders, 2) examined preclinical studies, animal models or cell culture models, 3) the involved signal pathway was CREB and its downstream regulators in psychiatric disorders, and 4) the treatment of antipsychotics in psychiatric disorders needed CREB involvement.

The following exclusion criteria were defined: 1) the publication time of studies was more than ten years, 2) the impact factor of studies was below three points, and 3) the research contents included other drug targets and illnesses.

Therefore, we decided to discuss our results according to psychiatric disorders, so firstly, we provided a classification of the disorders, then a paragraph regarding CREB distribution and structures, and CREB in psychiatric disorders, and finally, we discussed the role of CREB according to specific psychiatric illness (depression, schizophrenia, anxiety, OCD, and bipolar).

## 3 CLASSIFICATIONS OF PSYCHIATRIC DISORDERS

As mentioned above, psychiatric disorders are influenced by several factors. Hence, researchers divide psychiatric disorders into five main categories, including depression, anxiety disorder, obsessive-compulsive disorder, schizophrenia, and bipolar affective disorder, according to the clinical manifestation. For example, at the current medical level, psychiatric disorder classification is guided by sets of symptoms along with clinical course and CT scanning technology manifested by the patients, which has been applied to clinical practice [23]. The major factors that result in psychiatric disorders are mentioned in Table **1** [24-29].

Although the main factors can be used as the theoretical basis for the diagnosis of psychiatric disorders, they also have certain limitations. Therefore, it is necessary to figure out the underlying mechanisms of CREB in psychiatric disorders, which will be of great help to the treatment of psychiatric disorders in clinical guidance.

## 4 CREB: DISTRIBUTION AND STRUCTURE

CREB belongs to a member of the basic leucine zipper (bZIP) transcription factor gene family with a molecular weight of 43,000 kDa. The full-length sequence of CREB is made up of four functional domains, including Q1 basal transcriptional activity domain, kinase inducible domain (KID) containing protein kinases that can phosphorylate CREB, Q2/constitutive active domain (CAD) and bZIP domain for binding to DNA [30]. The function of CREB in the tissues of humans and mice is identical, which consists of 11 exons and three isoforms named α, β, and Δ that are produced through alternative splicing [31].

Several canonical signaling pathways are involved in CREB activation, including cAMP-protein kinase A (PKA)-CREB pathway, PI3K-Akt-GSK3β-CREB pathway, Ca^2+^-calmodulin kinases IV (CaMKIV)-CREB pathway, Ras-Raf-MAPK-p90RSK-CREB pathway, dopamine-mediated signaling, and D2 dopamine receptors (D2R) -CREB-BDNF-TrkB [32, 33].

**Fig. (4):**
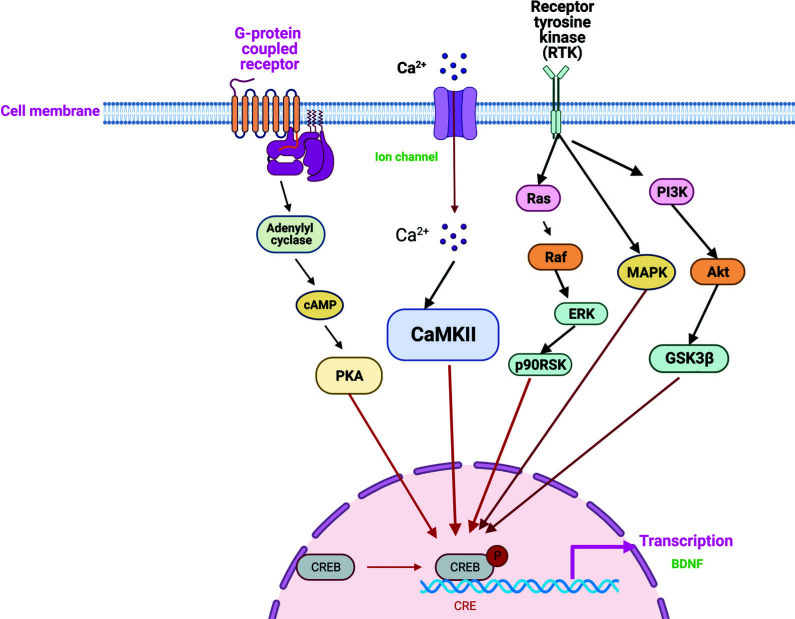
The CREB-mediated molecular network underlying psychiatric disorders. Image created by BioRender.com.

Recent studies have revealed that phosphorylation of CREB regulates gene transcription and then strengthens synaptic plasticity and neurodevelopment [34]. Therefore, it is reasonable to conclude that CREB is proposed to be involved in the disease process of psychiatric disorders (Fig. **4**).

## 5 THE ROLES OF CREB IN PSYCHIATRIC DISORDERS

### 5.1 CREB Signalling Cascade Plays Important Roles in Depression

Depression is the most common psychiatric disorder worldwide, which has a great impact on the quality of people's lives and strains the social welfare system. Depression has gradually become a public health issue. According to new data from WHO, over 792 million people worldwide are suffering from depression [35]; even worse, the incidence of suicide associated with depression has been increasing [36].

According to the Diagnostic and Statistical Manual of Mental Disorders, fifth edition, depression can be categorized into mild depression, moderate depression, and major depressive disorder (MDD) with different characteristics, such as the number and time of depressed mood or loss of interest or pleasure in activities of daily lives [37].

Many factors, including chronic stress, a functional deficit of monoaminergic neurotransmitters, overactivity of the HPA axis, and inflammation, are known to induce depressive symptoms in susceptible individuals [38]. At present, the treatments for depression have the following methods: psychotherapy for patients with mild-to-moderate depression in interpersonal relationship problems [39] and antidepressant medications, such as ketamine for moderate-to-severe depression with no apparent effect in psychotherapy. Despite massive improvements in fighting depression over the past few years, an antidepressant used in clinics has some imperfections in the side effect profile, large doses, and drug addiction [40]. Furthermore, the exact molecular pathogenesis of depression remains largely unknown.

**Fig. (5):**
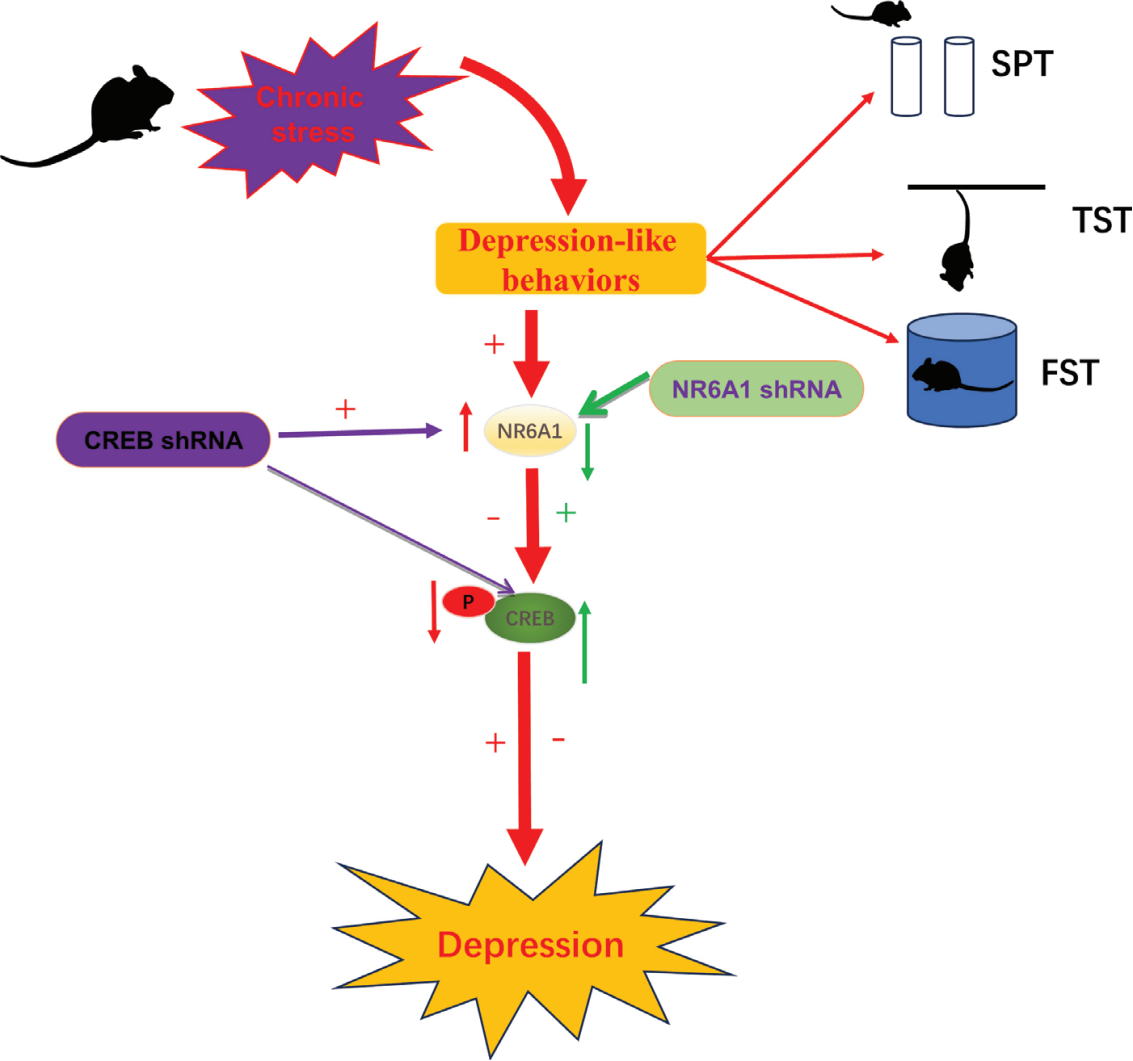
CREB and its downstream regulators relieved the depression-like behavior of rodent models.

Recent evidence has shown that the impairment of CREB and its signalling cascade participates in the pathogenetic process of depression simulated by animal models [20, 41]. The expression of CREB and its downstream regulators was noticeably decreased in rodent models of depression induced by chronic stress [42]. Moreover, inhibition of CREB expression exacerbated the depression-like behavior of normal mice (Table **2**) [43]. For example, Pingping *et al.* (2022) found that the expression of nuclear receptor subfamily 6, group A, member 1 (NR6A1) significantly increased in the hippocampus of chronic unpredictable stress (CUS)-induced mice and over-expression of NR6A1 contributed to the occurrence of depression-like behaviors and a significant reduction in the levels of phospho-CREB in normal mice. Furthermore, down-regulation of hippocampal NR6A1 alleviated depression-like behaviors of mice suffering from chronic stress by reversing the decreasing levels of phospho-CREB. Interestingly, the knockdown of CREB eliminated the anti-depressant effects of NR6A1 shRNA upon the increased consumption of sugar water in SPT (sucrose preference test) and decreased immobility time in TST (tail suspension test) and FST (forced swimming test) in CUS-induced mice (Fig. **5**) [20].

Fengfeng and *et al.* (2020) indicated that the expression of GPR39 was significantly increased in a hippocampal neuronal cell line of mice (HT-22) treated with 500 nM CORT at 6 h. However, the protein and mRNA expression of GPR39 and CREB in HT-22 treated with 1 μM, 10 μM, and 50 μM CORT were dramatically down-regulated at 12 h and 24 h as compared to the control group. Moreover, the expression levels of CREB protein were significantly increased in CORT-induced HT-22 cells treated with TC-G-1008, which was used to activate the expression of GPR39. The above results showed that GPR39 had protective effects on the neuronal injury in HT-22 cells of mice associated with the increased expression of the CREB signaling pathway [44].

**Fig. (6):**
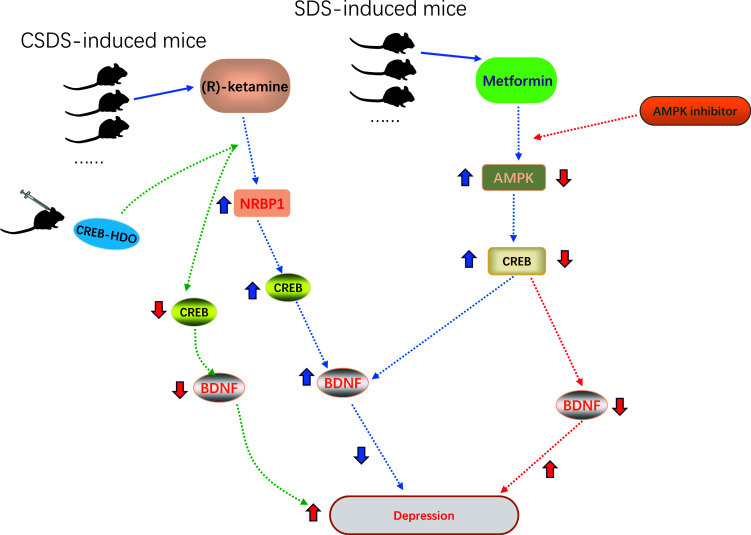
Antidepressants, such as (R)-ketamine and metformin, as a hypoglycemic agent exerted antidepressant effects by increasing the expression of the CREB signaling pathway.

Moreover, some studies suggest that a few drugs used in first-line treatment for depression exert antidepressant effects by promoting the expression of CREB. For example, the findings of a study by Wei Yao and Qianqian Cao research group showed that (*R*)-ketamine had antidepressant-like effects in CSDS-susceptible mice by activating novel extracellular signal-regulated kinase (ERK)-nuclear-receptor-binding protein 1 (NRBP1)-CREB-BDNF pathways in microglia. Interestingly, the Western blot reported that the expression of CREB was significantly decreased in the medial prefrontal cortex (mPFC) of mice after intracerebroventricular (i.c.v.) injection of CREB-HDO, and CREB-HDO markedly blocked antidepressant-like effects of (*R*)-ketamine in the behavior tests (FST and SPT) of CSDS-susceptible mice [41]. In addition to common anti-depression drugs, metformin as a hypoglycemic agent has been reported to have anti-depressant effects by activating the AMP-activated protein kinase (AMPK)/CREB signal pathway [45]. Specifically, metformin administration (200 mg/kg, i.g) mitigated the development of depression-like behaviours and improved structural plasticity in the hippocampus of social defeat stress (SDS) mice by increasing the expression of p-CREB/ CREB ratio (Fig. **6**).

Collectively, chronic stress leads to the development of depression-like behaviours in mice by decreasing the expression of the CREB signalling cascade. Improvement of CREB and its downstream regulators could help to relieve depressive symptoms.

### 5.2 CREB Participates in the Occurrence of Anxiety Disorder

Anxiety disorder forms part of a large group of prevalent psychiatric disorders and is often integrated with depression in similar symptoms, such as feelings of worry and fear, social phobia and avoidance behaviours. According to the statistics, the prevalence rates of anxiety disorders rank sixth among all mental and somatic illnesses worldwide that have a great influence on people's quality of life.

Anxiety disorder is a complex psychiatric disorder resulting from the interaction of multiple genetic variants with environmental factors. In clinical practice, psychotherapy and psychoactive drugs, including escitalopram, venlafaxine and pregabalin, are the first choice of therapeutic strategy for patients with anxiety disorder [46]. Although researchers have made some progress in the fight against anxiety disorder, many patients are not receptive to treatment or experience some side effects and have to stop taking daily medication. Therefore, it is necessary to find more effective treatment approaches.

Recent studies have reported that the CREB signaling pathway plays a vital role in anxiety disorder. It is well known that CREB, a critical regulator, aids in neuronal differentiation, survival and plasticity *via* activation of the BDNF-TrkB pathway (Table **3**) [47]. For example, Jiang *et al.* (2022) indicated that the CREB pathway mediated Tanshinone IIA to relieve anxiety-like behaviors in mice [13]. Specifically, administration of Tanshinone IIA (20 mg/ kg, i.p.) alleviated anxiety-like behaviors in model mice caused by conditioned fear (CF) and single-prolonged stress (SPS). Tanshinone IIA treatment increased the synaptic plasticity-related proteins in the hippocampus of CF and SPS-induced mice by enhancing the expression of the CREB/ BDNF/TrkB signalling pathway. Furthermore, blockage of CREB (a PKA inhibitor) abolished the antianxiety effects of TanIIA in model mice and reduced the levels of synaptic plasticity-related proteins in the hippocampus of mice treated with TanIIA. The result demonstrated that the CREB/BDNF/ TrkB signalling pathway in the hippocampus of mice has a great possibility of being involved in the antianxiety effect of TanIIA.

**Fig. (7):**
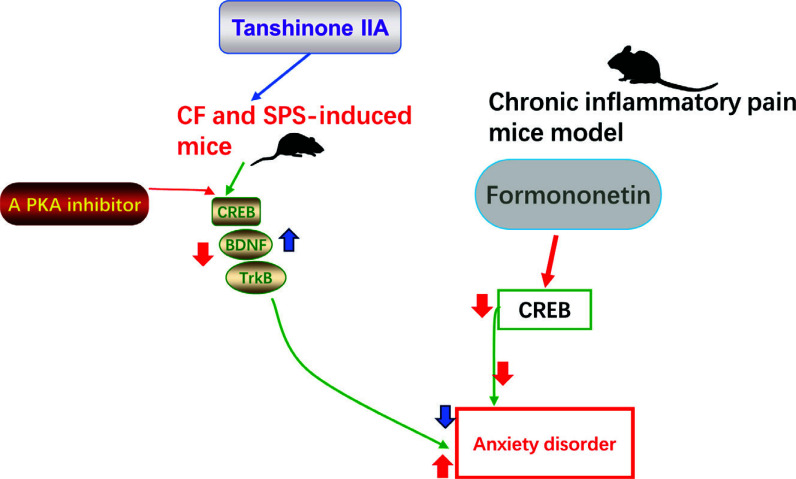
The CREB signaling pathway was involved in the development of anxiety disorder.

However, there are different opinions on the roles of CREB in anxiety disorder. For instance, the findings of a study by Wang *et al.* (2019) showed that Formononetin exerted anxiolytic effects in chronic inflammatory pain mice model due to the attenuation of neuronal hyperexcitability through the suppression of CREB expression in the basolateral amygdala of mice [48]. The results suggested that treatment with Formononetin (25 mg/kg) for 8 days relieved anxiety-like behaviour by inhibiting CREB signaling pathways (Fig. **7**). Moreover, the CREB gene is associated with early treatment response to escitalopram in anxiety disorder. Jun-Feng Yang and Shen Li demonstrated that CREB was involved in the antianxiety effects of escitalopram in patients with anxiety [49]. The CREB-BDNF pathway contributed to the improvement of clinical symptoms under escitalopram treatment for anxiety patients.

### 5.3 CREB Plays an Important Role in the Development of Schizophrenia

Schizophrenia is a severe mental illness with inferior rates of survival all over the world that imposes an enormous burden on medical and health services. The main clinical manifestations of patients with schizophrenia are delusions, hallucinations, social withdrawal, and cognitive dysfunction [50].

The aetiology of schizophrenia is multifactorial and mainly caused by gene susceptibility in individuals interacting with various environmental factors [51]. Research showed that the combination of genetic and environmental risk factors impaired neurotransmitter transmission and neural circuits [52]. At present, the main therapies for schizophrenia are pharmacotherapy, including atypical antipsychotics (clozapine, risperidone and aripiprazole), dopamine antagonists (pimozide), dopamine-serotonin antagonists (paliperidone), and dopamine-functionally selective (cariprazine), but these drugs have negative factors with slow efficacy, poor stability, and serious adverse side effects [53].

**Fig. (8):**
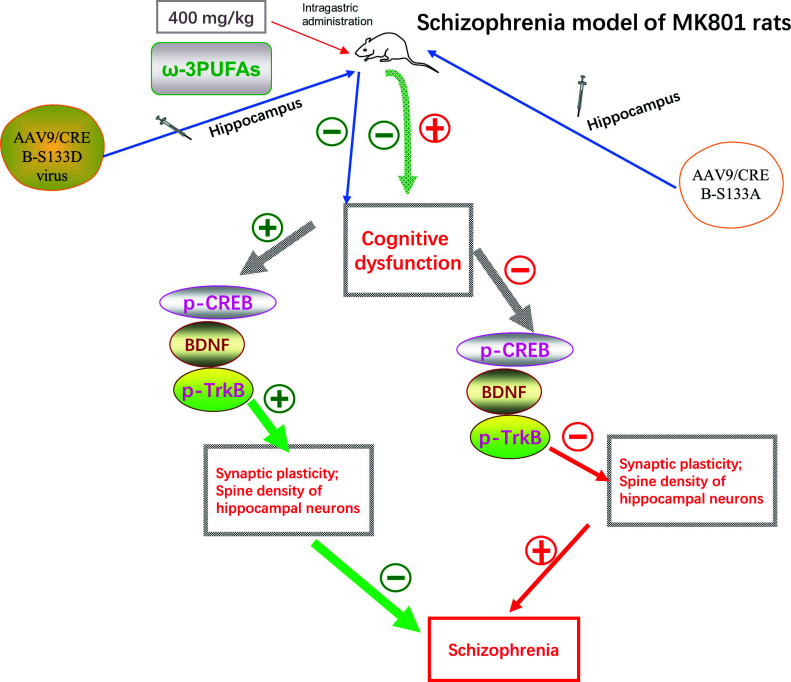
The deficit of CREB/BDNF/TrkB signaling impairs neurogenesis and is implicated in the development of schizophrenia.

However, recent research has shown that the CREB signalling pathway plays an important role in the pathophysiology of schizophrenia [54]. Dopamine, a brain neurotransmitter derived from hypothalamus neurons, is identified to participate in the pathology of schizophrenia for its hyperactive signal transduction. Dopamine might modulate CREB phosphorylation *via* activation of BDNF signaling [55]. For example, Jae Hoon Cheong reported that the activation of the dopamine pathway in the mesolimbic system of mice increased the levels of p-CREB and BDNF in the nucleus accumbens [56]. Furthermore, lower levels of CREB were observed in the brains of patients with schizophrenia (Table **4**) [57]. The results from a study by Guo *et al.* (2020) indicated that ω-3PUFAs alleviated schizophrenia-like behavioural alterations and improved synaptic dysfunctions in the schizophrenia model of MK801 rats. ω-3PUFAs restored neuronal damage in the hippocampus of MK801 rats *via* increasing the levels of p-CREB, BDNF, and p-TrkB. However, the rats' hippocampal injected with AAV9/CREB-S133A virus (a virus used to knockdown the expression of CREB) abolished the preventive effects of ω-3PUFAs on cognitive dysfunction, synaptic plasticity, and spine density of hippocampal neurons in MK801-induced mice of schizophrenia. Moreover, rats treated with AAV9/CREB-S133D virus improved cognitive dysfunction and dendritic morphology against schizophrenia (Fig. **8**) [58].

Interestingly, the BDNF-CREB signalling pathways were found to be involved in the pharmacological mechanism of antipsychotic treatment in schizophrenia [59]. The combination of haloperidol (1 mg/kg) and fluvoxamine (10 mg/kg) significantly increased the expression of CREB phosphorylation and BDNF in the hippocampus and frontal cortex of rats, and higher levels of CREB mRNA were detected after haloperidol-fluvoxamine combined treatment. In addition, haloperidol + fluvoxamine treatment improved CREB protein and mRNA expression levels in peripheral mononuclear cells (PMC) of schizophrenic patients after 3 and 6 weeks of therapy. CREB mRNA expression in PMC was significantly correlated with improved cognitive symptoms in patients with schizophrenia [59].

In summary, findings suggest a link between CREB and the pathophysiology of schizophrenia and may advance the development of effective drugs for this disease, thus replacing ineffective drugs.

### 5.4 CREB is Involved in the Development of Obsessive-compulsive Disorder

Obsessive-compulsive disorder (OCD) is a relatively common mental disorder with great harm to people's quality of life. The incidence rate of OCD is increasing yearly with increasing social pressure. According to statistics, regrettably, the symptoms of OCD are seen in adolescents that cause a lot of damage to physical and psychological health [60]. Patients with OCD usually exhibit signs of intrusive thoughts or images and repetitive actions. At the very beginning, people described the etiopathogenesis of OCD as automatism, but with the progress of science and technology, it is regarded as a neuropsychiatric disorder closely related to other psychiatric illnesses, such as depression and anxiety disorder that are mediated by specific neurotransmitters [61].

The possible organic causes, including genetic and environmental factors, trigger OCD symptoms. Meanwhile, environmental factors, such as stress and trauma, are widely regarded as the triggers behind the development of OCD that could alter gene expression [62]. Research reported that the levels of TNF-α, IL-6, noradrenalin, and serum cortisol were increased in patients with obsessive-compulsive disorder compared to controls [63]. The biological mechanism of OCD is the maladjustment of neurotransmitters, including glutamate, gamma-aminobutyric acid (GABA), dopamine and serotonin [64-66]. Nowadays, many first-line pharmacological treatments for OCD are developed based on the regulation of neurotransmitters, such as selective serotonin reuptake inhibitors (SSRIs, Fluvoxamine) and glutamatergic agents (memantine) [67]. Although appropriate pharmacotherapy significantly relieves clinical symptoms of patients with OCD at the initial stage of the disease, it is not effective for the remission of patients with moderate or severe OCD that requires long-term medication. Therefore, the development of new, more effective treatments for OCD is necessary.

**Fig. (9):**
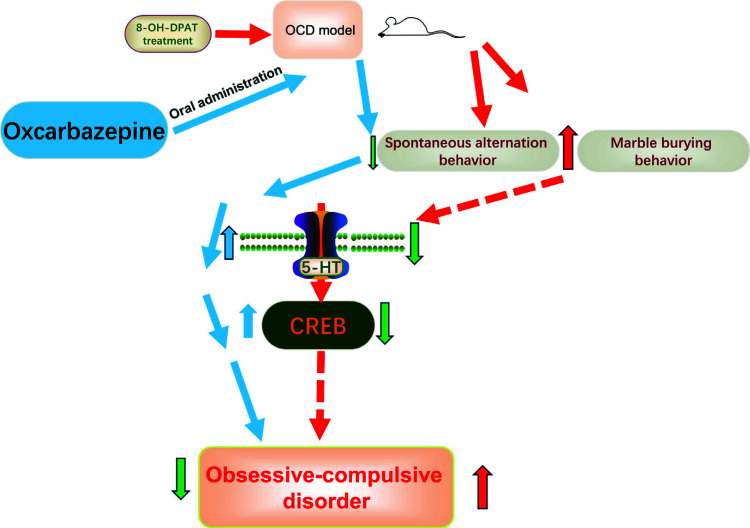
CREB participated in the anti-OCD effects of oxcarbazepine (OXC) in the OCD model of mice.

CREB is a member of the leucine zipper family of transcription factors, and the size of the protein is about 43 kDa. CREB has been demonstrated to mediate the effects of neurotransmitters and regulate many aspects of neuronal functioning, such as neuronal excitation, development, and long-term synaptic plasticity [68]. Recent studies have shown that CREB is involved in the pathogenesis of OCD and has neuroprotective effects against this disease. For example, CREB participated in the anti-OCD effects of oxcarbazepine (OXC) in the OCD model of mice [69]. OXC at the dosage of 20 mg/kg and 40 mg/kg significantly reduced the spontaneous alternation behaviour (SAB) and marble-burying behaviour (MBB) score in the OCD model of mice induced by 8-OH-DPAT treatment (2.8 mg/kg i.p.). OXC at a dose of 20 mg/kg restored the decrease in CREB caused by 8-OHDPAT in the frontal cortex of mice. In addition, the frontal cortical serotonin levels (5-HT) were increased after the 8-OHDPAT-induced mice being treated with oxcarbazepine. The results showed that the anti-OCD effects of OCX were possibly mediated *via* the increased levels of cortical 5-HT and CREB in the frontal cortex of mice (Fig. **9**). Rohbani *et al.* (2019) indicated that environmental factors, such as parental morphine, exposure before gestation increased the occurrence of OCD-like behaviour (grooming behaviour and marble burying) in the rats. The increased levels of CREB phosphorylation were observed in the offspring of morphine-abstinent mice as compared to normal mice [70]. The current results indicated that the higher levels of p-CREB in the nucleus accumbens (NAc) of rats might be involved in OCD simulated by morphine-addicted animals.

### 5.5 Roles of CREB Signaling in Bipolar Disorder

Bipolar disorder (BD) is a common neuropsychiatric disorder with a high morbidity rate. It has been estimated that the lifetime prevalence of BD is more than 4% of the global population and remains the sixth leading cause of disability [71]. It is surprising that the number of BD diagnoses has increased significantly in children and adolescents over the past few years. The clinical symptoms of patients with BD are recurrent mania, depression or a combination of both. The etiopathogenesis of BD is a result of the interaction between genetic and epigenetic factors with environmental influences [72]. In addition, the roles of monoamines (serotonin and dopamine) as the biological mechanisms of BD have been extensively studied, which provides a fundamental basis for the development of antipsychotic drugs [73]. In recent years, the therapeutically relevant mechanisms of action of lithium have drawn much attention from researchers. Pharmacological evidence shows that lithium can improve bipolar depressive symptoms through inhibiting glycogen synthase kinase-3 (GSK3) activity, including WNT/GSK3β/ β-catenin signaling, β-Arrestin-2/AKT/PP2A-GSK3 signaling, GSK3β/Collapsin response mediator protein 2 (CRMP2), and GSK3β/inositol monophosphatase (IMPase) [74]. In clinical practice, several treatments have been employed, including psychotherapies, such as behavioural activation and acceptance, drug intervention, such as placebo treatment and antidepressants, and instrumental therapies, such as vagus nerve stimulation and deep brain stimulation [75], but the main treatment is somatotherapy.

Despite these advances in the treatment of BD, many patients respond poorly to drug treatments. Moreover, these drugs can have side effects, with gaining weight, elevating lipids, raising blood pressure, and teratogenicity. Hence, it is urgent to develop better alternatives to improve the curative effect of existing treatments for BD. There is evidence that CREB signalling plays a mechanistic role in BD. CREB-regulated intracellular signalling pathway modulated by lithium is closely associated with the pathogenesis of BD [76, 77]. This means that CREB phosphorylation is an important target of lithium [78]. Meanwhile, there are numerous molecular targets in the downstream of CREB. Among them, the more important ones are BDNF and corticotropin release factor (CRF) [79]. Therefore, CREB is regarded as a central point of many signalling pathways in psychiatric disorders and plays an important role in synaptic plasticity and long-term memory formation (Table **5**) [80]. Alda *et al.* (2013) indicated that the abnormalities of CREB signal transduction were one of the most important susceptibility factors in patients with BD [81]. Compared to healthy subjects, pCREB levels were increased in patients with BD. No obvious differences in the level of CREB were observed between healthy subjects and patients with BD. However, the expression level of CREB was significantly improved in lymphoblasts from lithium-treated patients with BD as compared to control subjects. These results suggest that the up-regulation of p-CREB in lymphoblasts may be potential endophenotypes for patients with BD (Fig. **9**).

Odagaki *et al.* (2001) reported that the levels of p-CREB and CREB were elevated significantly in the prefrontal cortex of depressed suicide victims [82]. This finding was in agreement with previous studies. Gaspar *et al.* also confirmed that a 3.3-fold increase in the levels of CREB signalling was observed in fibroblasts from BD patients [83].

**Fig. (10):**
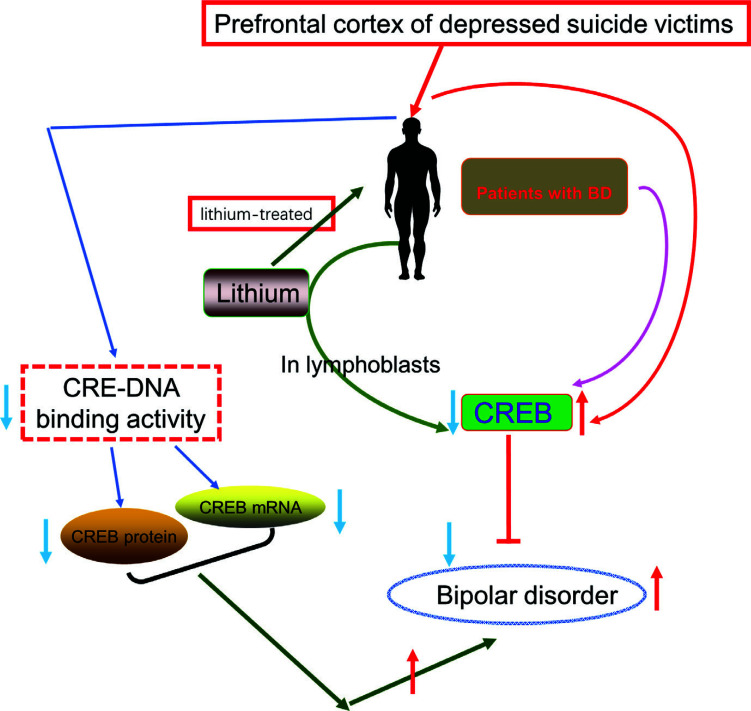
Abnormalities of CREB functions in patients were associated with the pathophysiology of BD illness.

However, other researchers did not hold the same view. Pandey *et al.* confirmed that the expression level of CREB was decreased in the dorsolateral prefrontal cortex (DLPFC) of postmortem patients with BD [84]. They suggested that the CRE-DNA binding activity detected by gel mobility shift assay was decreased significantly in the DLPFC, and cingulate gyrus (CG) samples of postmortem brains were obtained from patients subjected to BD. In addition, the reduced protein expression and mRNA levels of CREB were also observed in DLPFC and CG of the posthumous subjects who suffered from BD (Fig. **10**).

To sum up, abnormalities of CREB functions are associated with the pathophysiology of BD illness.

## CONCLUSION AND FUTURE PROSPECTS

**Fig. (11):**
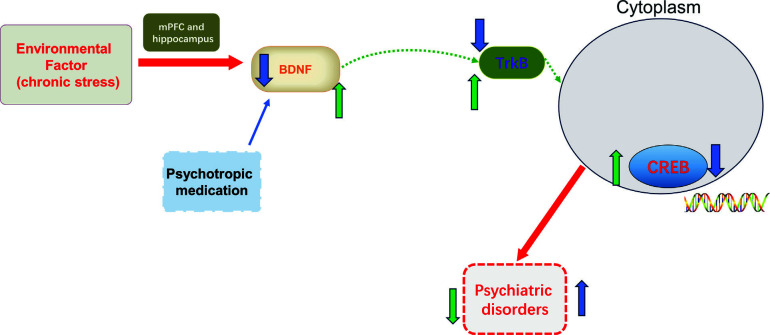
CREB and its downstream regulators participated in the antipsychotic effects of psychotropic medication.

With the rapid development of society, psychiatric disorders are becoming increasingly serious problems with high incidence rates, especially among adolescents. Due to the complex pathogenic factors and pathological processes, much effort has been spent on searching for effective treatment by researchers. In this review, we have demonstrated that the CREB signal pathway is involved in the development of psychiatric disorders, including depression, anxiety disorder, schizophrenia, obsessive-compulsive disorder, and bipolar disorder. Moreover, we also find powerful evidence that CREB and its downstream regulators participate in the antipsychotic effects of psychotropic medications as used in the clinical front line (Fig. **11**).

Patients with psychiatric disorders usually manifest as cognitive impairment, including a decline in psychological enduring capacity, attention, and capability to fix problems [85]. In addition to cognitive impairment, the pathogenesis of psychiatric disorders is associated with significant changes in brain structure, dysfunction of neurotransmitter systems, impaired neuroplasticity and neurogenesis, and neuroinflammation. Therefore, it is of great interest to identify molecular regulators that regulate lower cognitive function, neurodevelopment and nerve regeneration. Recent studies have revealed that CREB, a transcription factor that is implicated in multiple signalling pathways, modulated cognition and neuroplasticity in rodent models and was used to simulate psychiatric disorders in humans [33]. In this review, we found that the up-regulation of CREB facilitated performance in behavioural tasks for cognition in depression, anxiety disorder, schizophrenia and obsessive-compulsive disorder but resulted in the production of behaviours in bipolar disorder. Hence, we speculated that activation of CREB signalling had beneficial effects in the therapy of psychiatric disorders, while persistent CREB activation led to serious consequences.

It has been known that the signaling pathways involved in psychiatric disorders are multiple and complex. Numerous studies have demonstrated that interaction between CREB and BDNF enhances neuronal growth and synaptic transmission. Significantly, our review also indicates that the CREB-BDNF signal pathway plays important roles in the symptomatology, pathogenesis, and treatment of psychiatric disorders. The deficit of CREB/BDNF signalling impairs neurodevelopment and contributes to psychiatric disorders. A lot of CREB target genes have been demonstrated to be closely related to psychiatric disorders, such as BDNF, protein kinase B (Akt), and glycogen synthase kinase 3β (GSK3β) [86]. BDNF has been proven to be an important regulator of synaptic transmission and long-term potentiation (LTP) in the hippocampus of patients with neuropsychiatric disorders [87]. Research has proved that hippocampal CREB phosphorylation (p-CREB) promoted the expression of BDNF and alleviated depression-like behaviors in lipopolysaccharide-treated mice [88]. Thus, the CREB-BDNF system is a potential target for developing new pharmacotherapies for psychiatric disorders.

We also found that antipsychotic drugs exert their prompt clinical curative effects *via* activating CREB and its downstream target. The activation of CREB regulates gene transcription and translation in the hippocampus of mice [89]. The CREB-BDNF pathway contributes to neuronal survival, synaptic development and activity in the mPFC and hippocampus [90]. Thus, the increased levels of CREB and BDNF are important mechanisms underlying the clinically beneficial effect of antipsychotic drugs.

Moreover, neuroinflammation also plays an important role in the progression of psychiatric disorders [91]. CREB acts as a transcription factor that regulates the expression of genes and inhibition of inflammation in the pathophysiology of neuropsychosis [32]. In our review, we did not summarize the neuroinflammatory processes implicated in psychiatric disorders, and the mechanisms of how they promote the development of the disorders were limited. These results deserve more *in vitro* studies to define mechanisms in the future (Tables **2**-**5**) [92-109].

In summary, our findings reveal a vital role for CREB in relieving the behaviour characteristics of patients with psychiatric disorders. Targeting CREB signaling molecules promotes neurodevelopment in patients. However, it is worth pointing out that the increased expression of CREB is a risk factor for bipolar disorder, as data from the dorsolateral prefrontal cortex from postmortem patients suggests that pCREB levels are increased in healthy subjects who suffer from bipolar disorder. Therefore, the levels of CREB phosphorylation should be maintained at reasonable levels in the brains of normal individuals. With the in-depth study on CREB-related signaling pathways, CREB has the potential to become a more effective target for the therapy of psychiatric disorders, thus replacing existing ineffective drugs.

## Figures and Tables

**Fig. (3):**
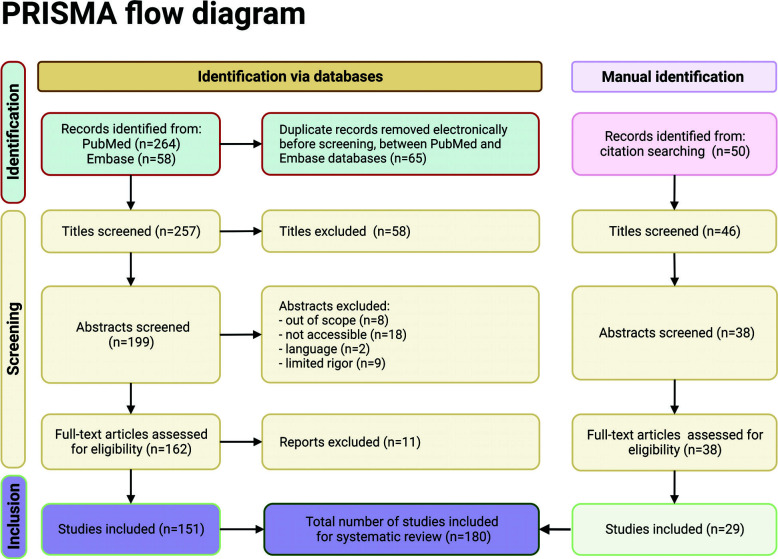
The screening process of articles on psychiatric disorders. Image created by BioRender.com.

**Table 1:** The major factors that result in psychiatric disorders.

**Classification**	**Internal Factors**	**External Factor**	**References**
Depression	Hypothalamic-Pituitary-Adrenal (HPA axis); Monoamine Neurotransmitter; Neuroinflammation	Social and environmental factors	[24]
Anxiety disorder	Genetic factors; Gut microbes; Dysregulated HPA axis signalling	Environmental factors	[25, 26]
Obsessive-compulsive disorder	Neural circuits; Neurological insults	Stressful or traumatic events	[27]
Schizophrenia	Neurotransmitters; Inflammation and Oxidative Stress	Stressful or traumatic events	[28]
Bipolar affective disorder	Genetics; Dysregulated HPA axis; Inflammatory disturbance	Environmental factors	[29]

**Table 2:** CREB plays a key role in depression pathogenesis.

**Models**	**Tissue or Serum**	**Expression ** **Level**	**Regulatory Role**	**Disease**	**Targets**	**References**
CUS-induced mice	Hippocampus of mice	Up-regulation	Alleviated depression	Depression	CREB-BDNF	[20]
CORT-induced hippocampal cells	Hippocampal cells (HT-22)	Down-regulation	Induced depression	Depression	GPR39-CREB-BDNF	[44]
CUMS-induced mice	Hippocampus of mice	Up-regulation	Alleviated depression	Depression	BDNF/ERK/CREB signaling pathway	[92]
Lipopolysaccharide (LPS)-induced mice	Hippocampus of mice	Up-regulation	Alleviated depression	Depression	pCREB/BDNF/PSD95/Synapsin1 signaling	[88]
Rotenone-induced mice	Hippocampus of mice	Up-regulation	Alleviated depression-like behaviors of mice	Depression	BDNF/TrkB/CREB	[93]
Methamphetamine induced depression	Hippocampus of rats	Down-regulation	Induced behavioral changes in adult rats	Depression	CREB/BDNF and Akt/GSK3 signaling pathway	[14]
“MCAO + isolation + CUMS” model of rats	Hippocampus of rats	Down-regulation	Induced depression	Depression	p-CREB/BDNF pathway	[94]
Chronic social defeat stress (SDS)-induced mice	Hippocampus of mice	Down-regulation	Induced depression-like behaviors of mice	Depression	AMPK/CREB	[95]
Traumatic brain injury (TBI)-induced rats	Nucleus accumbens (NAc) of rats	Overexpression	Mitigate TBI-induced depression	Depression	HO-1/CREB	[96]
CUMS-induced mice	Hippocampus of mice	Down-regulation	Induced depression-like behaviors of mice	Depression	CREB/BDNF	[97]

**Table 3:** CREB signaling pathway is involved in the development of anxiety disorder.

**Models**	**Tissue or Serum**	**Expression ** **Level**	**Regulatory Role**	**Disease**	**Targets**	**References**
CF and SPS-induced mice	Hippocampus of mice	Up-regulation	Alleviated anxiety-like behaviors	Anxiety	CREB/BDNF/TrkB	[13]
Chronic inflammatory pain mice model	Basolateral amygdala of mice	Down-regulation	Alleviated anxiety-like behaviors	Anxiety	NMDA receptors and CREB	[48]
Peripheral venous blood samples from patients	Blood samples	Up-regulation	Alleviated anxiety-like behaviors	Anxiety	CREB-BDNF	[49]
A chronic alcohol exposure rat model	mPFC of rats	Up-regulation	Induced anxiety-like behavior	Anxiety	PI3K-AKT-GSK3β-CREB	[98]
Lipopolysaccharide-induced anxiety-like behaviour in rats	mPFC of rats	Up-regulation	Alleviated anxiety-like behaviors	Anxiety	BDNF/CREB	[99]
Chronic restraint stress (CRS)-induced rats	mPFC and hippocampus of rats	Up-regulation	Alleviated anxiety-like behaviors	Anxiety disorders	cAMP/PKA/CREB/ BDNF	[100]
Mice exposed to an anxiety-related environment	Hippocampus of mice	Down-regulation	Induced anxiety-like behaviors	Anxiety disorders	ERK-BDNF-CREB	[101]

**Table 4:** CREB is involved in the signaling pathways, leading to the pathogenesis and therapy of schizophrenia.

**Models**	**Tissue or Serum**	**Expression ** **Level**	**Regulatory Role**	**Disease**	**Targets**	**References**
Schizophrenia model of MK801 rats	Hippocampus of rats	Up-regulation	Alleviated schizophrenia	Schizophrenia	CREB/BDNF/TrkB Signal	[58]
Juvenile MAM mice model of schizophrenia	PFC of mice	Down-regulation	Induced schizophrenia	Schizophrenia	AKT/CREB/NR2B	[102]
Schizophrenia model of MK801 mice	Hippocampus and mPFC of mice	Up-regulation	Alleviated schizophrenia	Schizophrenia	p-CREB, BDNF and its receptor Trk-B	[103]
Patients with schizophrenia	Peripheral blood of patients	Down-regulation	Induced schizophrenia	Schizophrenia	BDNF, PI3K, AKT, and CREB	[57]
A mouse model of schizophrenia	Hippocampus of mice	Down-regulation	Induced schizophrenia	Schizophrenia	BDNF/CREB	[104]
Schizophrenia model of MK801 rats	Hippocampus of rats	Up-regulation	Alleviated schizophrenia	Schizophrenia	ERK1/2-CREB	[105]
Primary cultures of murine cortical and hippocampal neurons	Murine neuron primary cell cultures	Down-regulation	Alleviated schizophrenia	Schizophrenia	Neurotrophin VGF and PI3K/AKT/CREB pathway	[106]

**Table 5:** Abnormal expression of CREB participates in the pathophysiology of BD.

**Models**	**Tissue or Serum**	**Expression ** **Level**	**Regulatory Role**	**Disease**	**Targets**	**References**
Patients with BD	Lymphoblasts of patients	Up-regulation	Induced BD	Bipolar disorder	CREB Signal	[81]
Patients with BD	mPFC of depressed suicide victims	Increased significantly	Induced BD	Bipolar disorder	CREB	[82]
Patients with BD	Fibroblasts of subject	Up-regulation	Induced BD	Bipolar disorder	CREB	[83]
Patients with BD	DLPFC and CG of patients	Low expression	Induced BD	Bipolar disorder	CREB	[84]
Rat models of BD	Cell nuclei from the hippocampus of Rats	Down-regulation	Alleviated BD	Bipolar disorder	p-CREB/CaMKIV	[107]
Ouabain-induced rat models of BD	Frontal cortex and hippocampus of the rats	Down-regulation	Alleviated BD	Bipolar disorder	BDNF-TrkB-CREB	[108]
Hamster insulinoma tumor cells (HIT-T15)	Cell cultures	Up-regulation	Alleviated BD	Bipolar disorder	CREB-CBP-CRTC	[109]

## LIST OF ABBREVIATIONS

BD: Bipolar Disorder
BDNF: Brain-derived Neurotrophic Factor
CAD: Constitutive Active Domain
CF: Conditioned Fear
CUS: Chronic Unpredictable Stress
GSK3: Glycogen Synthase Kinase-3
KID: Kinase Inducible Domain
LTP: Long-term Potentiation
MBB: Marble-burying Behaviour
MDD: Major Depressive Disorder
OCD: Obsessive-compulsive Disorder
PMC: Peripheral Mononuclear Cells
SDS: Social Defeat Stress
SPS: Single-prolonged Stress

## STANDARDS OF REPORTING

PRISMA guidelines and methodologies were followed in this study.

## SUPPLEMENTARY MATERIAL

PRISMA checklist is available as supplementary material on the publisher’s website along with the published article.
